# The Role of Immature Granulocyte Count and Percentage as Novel Indicators of Systemic Inflammation in Plaque Psoriasis

**DOI:** 10.3390/jcm15166133

**Published:** 2026-08-07

**Authors:** Ali Osman Metintaş, Hüseyin Yaman, Leyla Baykal Selçuk, Arzu Ferhatosmanoğlu, Mahir Dığış, İbrahim Etem Arıca, Deniz Aksu Arıca, Ömer Faruk Saz

**Affiliations:** 1 Dermatology Clinic, Ağrı Training and Research Hospital, Agri 04100, Türkiye; 2 Department of Biochemistry, Farabi Hospital, Faculty of Medicine, Karadeniz Technical University, Trabzon 61080, Türkiye; huseyinyaman28@gmail.com; 3 Department of Dermatology, Farabi Hospital, Faculty of Medicine, Karadeniz Technical University, Trabzon 61080, Türkiye; lb_leyla@hotmail.com (L.B.S.); arzuferhatosman@gmail.com (A.F.); dretemarica@gmail.com (İ.E.A.); drdenizaksu@gmail.com (D.A.A.); 4 Department of Dermatology, Faculty of Medicine, Firat University, Elazig 23119, Türkiye; mdigis@firat.edu.tr; 5 Department of Biochemistry, 25 December State Hospital, Gaziantep 27090, Türkiye; faruksaz@outlook.com

**Keywords:** biomarkers, granulocytes, inflammation, psoriasis, therapy

## Abstract

**Background/Objective:** Psoriasis is a chronic immune-mediated inflammatory disease with systemic involvement. Immature granulocytes (IGs) are potential inflammatory markers, but their role in psoriasis is unclear. We evaluated immature granulocyte count (IG#) and percentage (IG%) in plaque psoriasis, their associations with disease severity and treatment response, and their ability to distinguish patients from healthy controls. **Methods:** This retrospective study included 85 adults with plaque psoriasis and 85 healthy controls. Severity was assessed by the Psoriasis Area and Severity Index (PASI). IG#, IG%, and inflammatory markers were measured before treatment and after at least 3 months of systemic therapy. Group comparisons, correlations, and receiver operating characteristic (ROC) analyses were performed. **Results:** Pre-treatment IG# and IG% were higher in patients than in controls (both *p* < 0.001). IG# decreased after treatment (*p* = 0.004), whereas IG% did not (*p* = 0.072). Despite clinical improvement, post-treatment IG#, IG%, and C-reactive protein remained higher than controls. Pre-treatment IG# correlated weakly with PASI (r = 0.24, *p* = 0.020), whereas other PASI–IG correlations were non-significant. Discriminatory performance was modest for IG# (AUC = 0.697) and IG% (AUC = 0.658). **Conclusions:** IG# and IG% are elevated in plaque psoriasis. IG# decreases with treatment and may be a potential marker of inflammatory changes, while the persistence of elevated IG# and IG% levels after treatment relative to healthy controls may reflect residual inflammation. However, weak associations with disease severity and modest discriminatory performance do not support their use as standalone clinical tools. As these parameters are routinely available from complete blood counts, they may complement clinical assessment. Further prospective studies are required to establish their clinical relevance.

## 1. Introduction

Psoriasis is a common, chronic, immune-mediated inflammatory disease affecting approximately 1.5–2% of the general population [1]. Although primarily characterized by erythematous, scaly plaques of the skin, psoriasis is now recognized as a systemic inflammatory disorder associated with multiple comorbidities, including psoriatic arthritis, metabolic syndrome, and cardiovascular disease [2]. The systemic inflammatory burden underlying psoriasis plays a crucial role in both disease severity and long-term complications. In this context, the exposome encompasses the cumulative internal and external exposures encountered throughout life, including lifestyle, metabolic, infectious, and environmental factors that may influence the onset and course of psoriasis. By modulating immune pathways and systemic inflammatory activity, these factors may shift the balance toward disease exacerbation when additional triggers are encountered [3]. This concept also provides a rationale for investigating changes in circulating inflammatory biomarkers following systemic anti-inflammatory treatment.

Assessment of disease severity is essential for therapeutic decision-making and monitoring treatment response. The Psoriasis Area and Severity Index (PASI) is one of the most widely used and validated scoring systems in adult plaque psoriasis and serves as a standard tool in both clinical practice and research settings [4]. However, clinical scoring systems alone may not fully reflect systemic inflammatory activity. Therefore, increasing attention has been directed toward identifying reliable, easily accessible laboratory biomarkers that may assist in evaluating disease activity and treatment response.

Immature granulocytes (IGs), which can be detected through automated complete blood count analysis, have emerged as novel markers of systemic inflammation. Automated hematology analyzers enable the routine quantification of immature granulocytes as part of the automated leukocyte differential, facilitating their evaluation as potential biomarkers in inflammatory diseases [5,6,7]. Neutrophils, the predominant circulating granulocyte population, play a well-established role in psoriasis pathogenesis [8]. Their accumulation within psoriatic lesions contributes to characteristic histopathological features, including Munro microabscesses, while neutrophil-derived cytokines, granular proteins, reactive oxygen species, and neutrophil extracellular traps (NETs) further amplify the IL-23/IL-17-driven inflammatory response [8]. Elevated IG count (IG#) and percentage (IG%) have been reported in various inflammatory and infectious conditions, including sepsis and acute inflammatory disorders, sometimes increasing earlier than conventional markers such as C-reactive protein (CRP) and leukocyte count [9,10,11,12,13]. Although the pathogenic role of mature neutrophils in psoriasis has been well established, the specific role of circulating immature granulocytes has not yet been elucidated, and evidence regarding their clinical significance in chronic inflammatory dermatologic diseases, particularly psoriasis, is scarce. Accordingly, IG# and IG% may represent potential peripheral biomarkers of systemic inflammatory activity rather than established mediators of psoriasis pathogenesis. Therefore, the present study aimed to evaluate the relationship between IG# and IG% and disease severity assessed by PASI in patients with plaque psoriasis. Additionally, we sought to compare these parameters with healthy controls and to investigate their potential utility in monitoring treatment response.

## 2. Materials and Methods

This single-center, retrospective observational study included patients diagnosed with plaque-type psoriasis between January 2017 and December 2024. A total of 85 adult patients who had been clinically and/or histopathologically diagnosed with plaque psoriasis and received systemic treatment (either conventional systemic agents or biologic therapies) were enrolled. Inclusion criteria were age ≥ 18 years; clinical and/or histopathological diagnosis of psoriasis; absence of active infection at the time of laboratory evaluation; not being pregnant or in the lactation period; and no history of chronic systemic disease. Patients with psoriatic arthritis (PsA) were not excluded; the presence of psoriatic arthritis was recorded and evaluated as a categorical variable (present/absent). The control group consisted of 85 age- and sex-comparable individuals without any known systemic or inflammatory disease who underwent routine laboratory testing, including complete blood count, erythrocyte sedimentation rate (ESR), and CRP, during general medical evaluation.

Demographic data (age, sex) and clinical characteristics were obtained from medical records. Disease severity was assessed using the PASI, and pre-treatment and post-treatment PASI scores were recorded. Pre-treatment values were defined as measurements obtained immediately before the initiation of systemic therapy. Post-treatment values were defined as measurements obtained at either the 3- or 6-month routine follow-up visit, after at least 3 months of continuous systemic treatment, when a ≥75% reduction from baseline PASI (PASI 75) had been achieved and no active infection was present. When the 3-month assessment did not meet these criteria because of active infection or failure to achieve PASI 75, the 6-month assessment was used. Hematological parameters obtained from routine automated complete blood count analysis at the corresponding time points were documented, including IG#, IG%, neutrophil count, lymphocyte count, and platelet count. Complete blood count analyses were performed in the hospital’s central laboratory using an automated hematology analyzer (Sysmex XN-1000, Sysmex Corporation, Kobe, Japan). IG# and IG% were generated as part of the automated differential leukocyte analysis performed by the analyzer. All laboratory analyses were performed according to the manufacturer’s recommendations using the laboratory’s routine internal and external quality control procedures. In addition, ESR and CRP levels were recorded. The neutrophil-to-lymphocyte ratio (NLR) and systemic immune-inflammation index (SII) were calculated using standard formulas. Comparisons were performed between the psoriasis and control groups in terms of immature granulocyte parameters as well as other inflammatory markers (ESR, CRP, NLR, and SII). Within the psoriasis group, pre-treatment and post-treatment values were compared. Correlation analyses were conducted to evaluate the relationship between PASI scores and immature granulocyte parameters, as well as other inflammatory indices.

### 2.1. Statistical Analysis

Statistical analyses were performed using SPSS software version 25.0 (IBM Corp., Armonk, NY, USA), while ROC curve analyses were conducted using MedCalc^®^ Statistical Software version 23.6.5 (MedCalc Software Ltd., Ostend, Belgium). Continuous variables were expressed as mean ± standard deviation (SD) for normally distributed data or as median (25th–75th percentiles) for non-normally distributed data. Categorical variables were presented as frequencies and percentages (%). The normality of continuous variables was assessed using the Kolmogorov–Smirnov test. For comparisons between independent groups, Student's *t*-test was used for normally distributed variables, whereas the Mann–Whitney U test was applied for non-normally distributed variables. For within-group comparisons of pre-treatment and post-treatment values, the paired *t*-test was used for normally distributed variables and the Wilcoxon signed-rank test for non-normally distributed variables. Categorical variables were compared using the chi-square test. Correlations between parameters were evaluated using Pearson or Spearman correlation analysis, as appropriate. Receiver operating characteristic (ROC) curve analyses were performed to evaluate the ability of pre-treatment IG# and IG% to differentiate patients with psoriasis from healthy controls. Areas under the ROC curves (AUCs) with 95% confidence intervals (CIs) were calculated. Optimal cut-off values were determined using the Youden index, and the corresponding sensitivity and specificity values with 95% CIs were reported. A *p*-value of <0.05 was considered statistically significant.

### 2.2. Ethics Committee Approval

Ethical approval was obtained from the Institutional Ethics Committee (protocol no. 2024/239, 7 March 2025; approval no. 24237859-164).

## 3. Results

A total of 85 patients with plaque psoriasis (51 males, 34 females) and 85 healthy controls (52 males, 33 females) were included in the study. There was no statistically significant difference between the groups in terms of sex distribution (*p* = 0.880). The mean age was 39 ± 13.7 years in the psoriasis group and 39.5 ± 12.4 years in the control group, with no significant difference observed (*p* = 0.760). Therefore, the two groups were comparable regarding age and sex.

When pre-treatment psoriasis patients were compared with the control group, both IG# and IG% were significantly higher in the psoriasis group (both *p* < 0.001). No significant difference was found in ESR between the groups (*p* = 0.080). However, CRP, white blood cell (WBC) count, and neutrophil count were significantly elevated in psoriasis patients compared to controls (all *p* < 0.001). Additionally, NLR and SII were significantly higher in the psoriasis group (both *p* < 0.001) (Table 1).

**Figure 1:**
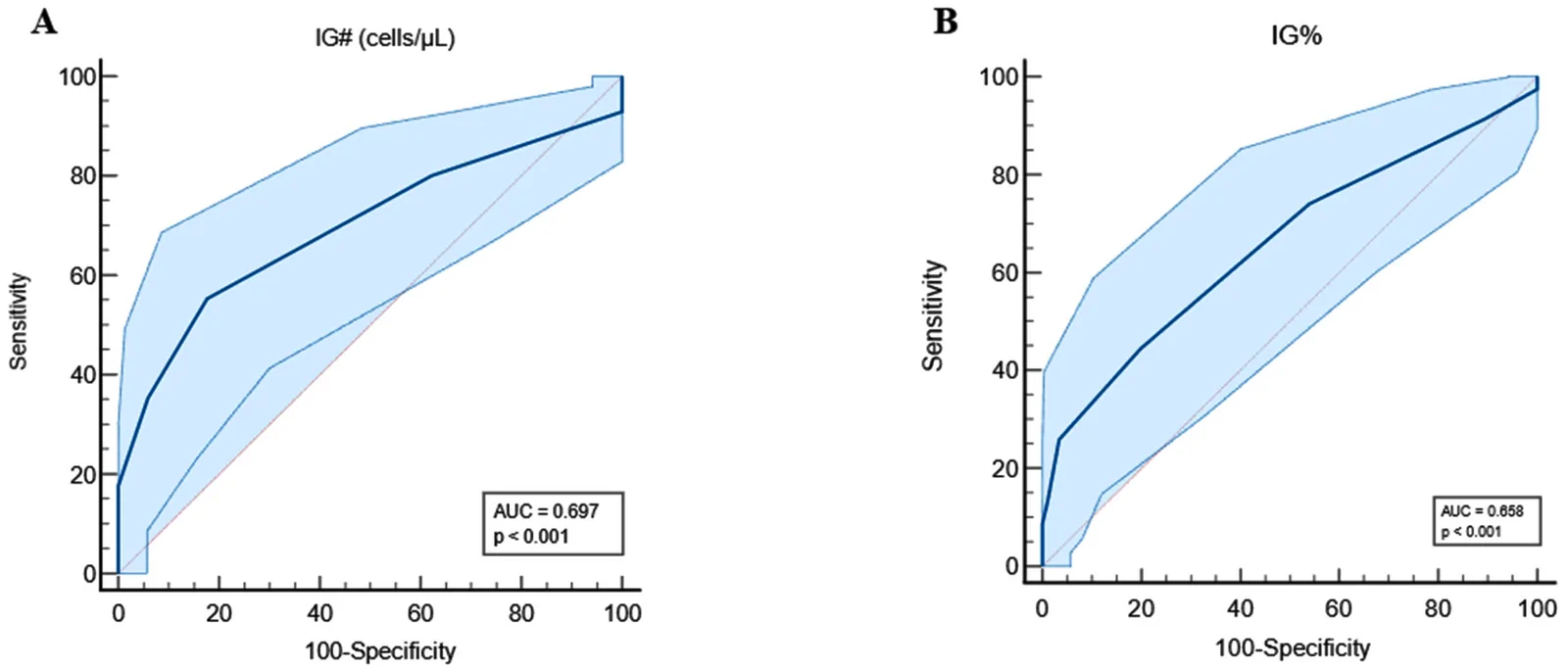
Receiver operating characteristic curves of pre-treatment immature granulocyte parameters for differentiating patients with plaque psoriasis from healthy controls. (**A**) Immature granulocyte count (IG#); (**B**) immature granulocyte percentage (IG%). The dark blue line represents the ROC curve, the shaded blue area represents the 95% confidence interval. The diagonal line represents the line of no discrimination. AUC, area under the curve; IG#, immature granulocyte count; IG%, immature granulocyte percentage.

ROC curve analysis showed modest discriminatory performance for pre-treatment IG# (AUC = 0.697, 95% CI: 0.622–0.765; *p* < 0.001) and IG% (AUC = 0.658, 95% CI: 0.581–0.730; *p* < 0.001) in differentiating patients with psoriasis from healthy controls. The ROC curves are presented in Figure 1, while the optimal cut-off values and corresponding sensitivity and specificity estimates are provided in Table 2.

A statistically significant reduction in PASI scores was observed after treatment (*p* < 0.001). Regarding inflammatory parameters, IG# showed a significant decrease following treatment (*p* = 0.004), whereas the change in IG% did not reach statistical significance (*p* = 0.072). CRP levels decreased numerically from 3.25 (1.48–6.33) mg/L before treatment to 2.8 (1.4–5.3) mg/L after treatment; however, this change did not reach statistical significance (*p* = 0.160). No significant difference was observed in ESR between pre-treatment and post-treatment measurements (*p* = 0.280). In contrast, both NLR and SII demonstrated a statistically significant reduction after treatment (both *p* < 0.001) (Table 3).

When post-treatment psoriasis patients were compared with healthy controls, IG# (*p* = 0.005) and IG% (*p* = 0.018) remained significantly higher in the psoriasis group. No significant difference was observed in ESR between the groups (*p* = 0.540). In contrast, CRP levels were significantly higher in post-treatment psoriasis patients compared to controls (*p* = 0.007). However, no statistically significant differences were found in NLR (*p* = 0.440) or SII (*p* = 0.520) between the two groups (Table 4).

**Figure 2:**
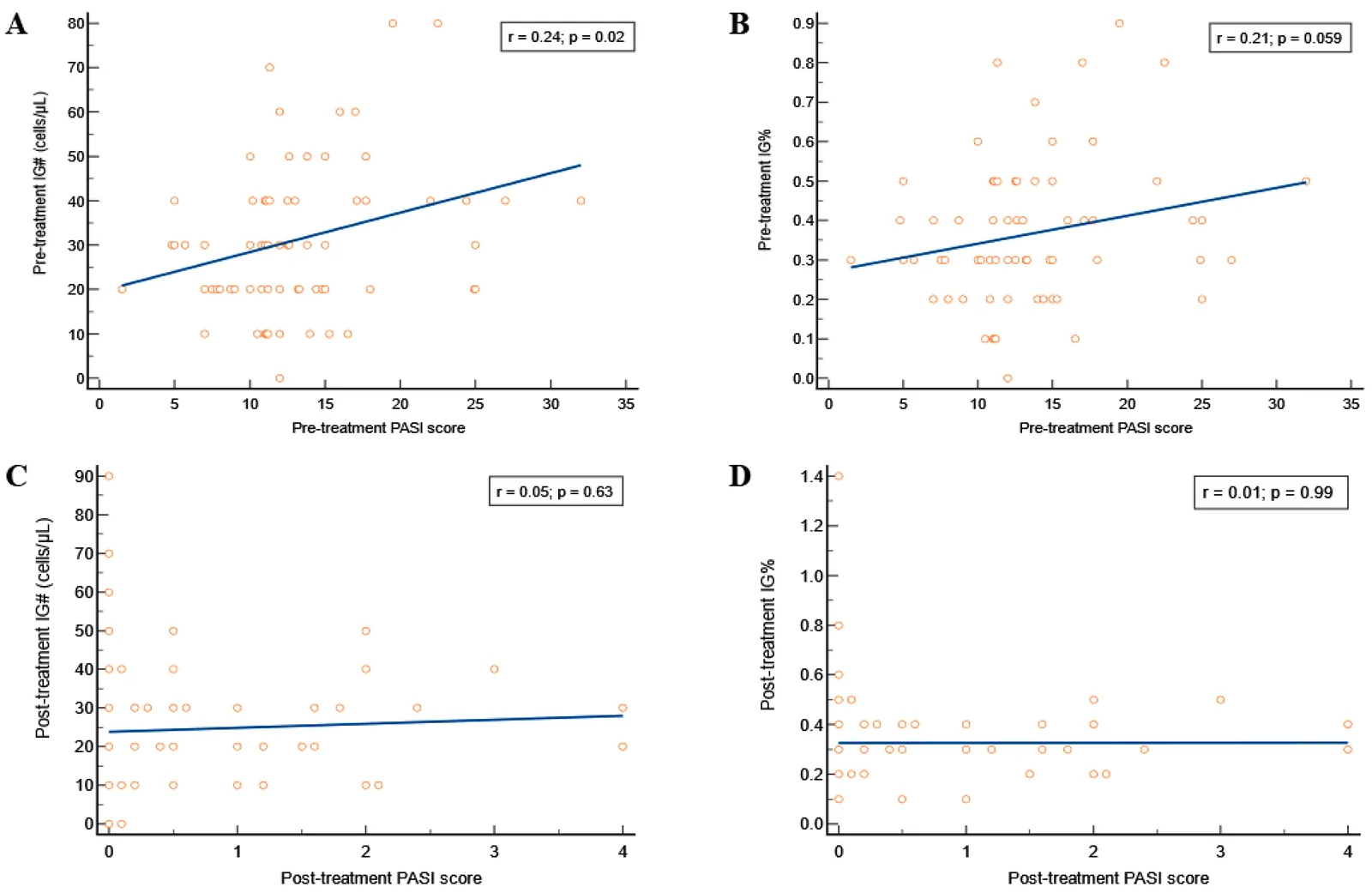
Correlations between PASI scores and immature granulocyte parameters before and after systemic treatment. (**A**) Correlation between pre-treatment PASI score and pre-treatment IG#. (**B**) Correlation between pre-treatment PASI score and pre-treatment IG%. (**C**) Correlation between post-treatment PASI score and post-treatment IG#. (**D**) Correlation between post-treatment PASI score and post-treatment IG%. Correlations were assessed using Spearman’s rank correlation analysis. The circles represent individual observations. The solid lines represents the fitted trend lines. PASI, Psoriasis Area and Severity Index; IG#, immature granulocyte count; IG%, immature granulocyte percentage.

A weak but statistically significant correlation was observed between pre-treatment PASI scores and pre-treatment IG# (r = 0.24, *p* = 0.020). No statistically significant correlations were observed between pre-treatment PASI scores and pre-treatment IG% (r = 0.21, *p* = 0.059), post-treatment PASI scores and post-treatment IG# (r = 0.05, *p* = 0.630), or post-treatment PASI scores and post-treatment IG% (r = 0.01, *p* = 0.990) (Figure 2). Age showed a weak negative correlation with post-treatment PASI scores (r = −0.22, *p* = 0.041). No significant associations were detected between age and pre-treatment PASI, nor with IG# or IG% at either the pre-treatment or post-treatment assessments (all *p* > 0.05) (Figure S1). When patients were stratified by sex, no statistically significant differences were observed between male and female patients in terms of pre-treatment or post-treatment PASI, IG#, or IG% values (all *p* > 0.05) (Table 5).

Among the 85 patients with psoriasis, 22 (25.9%) had concomitant PsA, while 63 (74.1%) did not. In the PsA group, there were 11 males and 11 females, whereas the non-PsA group consisted of 40 males and 23 females. The mean age was 42.2 ± 11.7 years in patients with PsA and 37.8 ± 14.3 years in those without PsA. There were no statistically significant differences between the groups in terms of sex distribution or age (*p* = 0.270 and *p* = 0.150, respectively), indicating that the groups were comparable in these respects. When pre-treatment parameters were compared between patients with and without PsA, no statistically significant differences were observed across any of the evaluated variables. Similarly, post-treatment comparisons revealed no significant differences between the groups for most parameters; however, ESR was found to be significantly higher in patients with PsA compared to those without PsA (*p* = 0.015).

## 4. Discussion

In this study, we assessed the role of IG# and IG% as indicators of systemic inflammation in patients with plaque psoriasis. Our findings revealed that both IG# and IG% were significantly elevated in psoriasis patients compared to healthy controls prior to treatment, thereby supporting their association with systemic inflammatory burden. Furthermore, IG# exhibited a significant reduction following systemic therapy, whereas IG% did not demonstrate a significant change. Notably, IG#, IG% and CRP levels remained significantly higher in post-treatment patients compared to controls, while NLR and SII normalized after treatment. These findings suggest that immature granulocytes, particularly IG#, may reflect both disease activity and residual inflammation in psoriasis.

IGs are early-stage myeloid cells released from the bone marrow into peripheral circulation during inflammatory conditions and are considered markers of innate immune activation [14]. Their presence in peripheral blood reflects bone marrow stimulation and an ongoing inflammatory response [14]. The mobilization of IGs from the bone marrow into the peripheral circulation, rather than representing a simple stress response such as demargination, reflects “emergency granulopoiesis” driven by systemic inflammation and regulated by cytokines such as type I interferons and granulocyte–macrophage colony-stimulating factor (GM-CSF) [15,16]. Neutrophil activation, which plays a central role in the pathogenesis of psoriasis, is closely linked to systemic inflammatory processes, and IGs may represent an extension of this neutrophil-driven response. Previous studies have demonstrated that IG levels increase in a wide range of infectious and inflammatory conditions, including sepsis, pulmonary embolism, acute appendicitis and fibromyalgia [10,17,18,19,20]. More recently, IG parameters have also been investigated in autoimmune and chronic inflammatory diseases such as pemphigus, familial Mediterranean fever, ankylosing spondylitis and rheumatoid arthritis, where they have been associated with disease activity and systemic inflammatory burden [21,22,23,24,25]. However, to the best of our knowledge, the role of IGs has not been adequately evaluated in chronic inflammatory dermatologic diseases such as psoriasis, and our findings contribute to the emerging evidence in this field.

Although ROC analyses showed that both IG# and IG% differentiated patients with psoriasis from healthy controls better than chance, their discriminatory performance was modest. IG# showed a numerically higher AUC than IG%, but its sensitivity remained limited despite relatively high specificity. These findings indicate that IG parameters should not be considered standalone diagnostic tools. Instead, they may provide complementary information regarding inflammatory status when interpreted together with clinical assessment and other laboratory findings.

Psoriasis is increasingly recognized as a systemic inflammatory disease involving complex interactions between innate and adaptive immune responses, and there has been growing interest in identifying reliable hematological markers to reflect disease activity, prognosis, and treatment response [26]. Neutrophil activation and migration play a key role in disease pathogenesis, and markers reflecting neutrophil dynamics have been investigated in recent years [26]. Recent meta-analyses have demonstrated that complete blood count–derived inflammatory indices such as NLR and SII are significantly elevated in patients with psoriasis compared to healthy controls and may correlate with disease severity [27]. In addition, several studies have reported that these parameters decrease following systemic or biological therapies, suggesting their potential utility in monitoring treatment response [28,29]. Consistent with previous studies, we observed elevated NLR and SII levels in psoriasis patients prior to treatment, indicating increased systemic inflammation. However, unlike IG#, both NLR and SII decreased significantly after treatment and were no longer different from controls. This finding suggests that while conventional inflammatory indices may reflect acute or reversible inflammatory changes, IGs may provide additional information regarding persistent or subclinical inflammation.

One of the notable findings of our study is that IG# decreased significantly following treatment, indicating its potential utility in monitoring treatment response. In contrast, IG% did not show a statistically significant change, suggesting that absolute immature granulocyte count may be a more sensitive and reliable parameter than percentage values in reflecting inflammatory dynamics. This discrepancy may be explained by the influence of total leukocyte count on percentage-based measurements, whereas absolute counts may better represent true changes in granulocyte production and release.

Another important observation is the persistence of elevated IG# and CRP levels in the post-treatment group compared to healthy controls, despite significant clinical improvement as reflected by PASI scores. CRP levels decreased numerically following systemic treatment, although this within-patient change did not reach statistical significance. Thus, while treatment was associated with a modest reduction in CRP, post-treatment levels remained higher than those observed in healthy controls. This pattern may indicate that improvement in cutaneous disease does not necessarily coincide with complete resolution of systemic inflammatory activity, as residual CRP elevation has also been reported in clinically improved patients with psoriasis [30]. Such subclinical inflammation may contribute to the increased risk of long-term comorbidities associated with psoriasis, including cardiovascular disease and metabolic disorders. However, the persistence and variability of CRP should be interpreted cautiously because CRP may reflect cardiometabolic factors, including adiposity, metabolic syndrome, hypertension, and overall cardiovascular risk, in addition to psoriasis-related inflammation [31]. Although patients with active infection and known chronic systemic diseases were excluded, BMI and smoking status were not systematically available and could not be evaluated. Heterogeneity in systemic treatment modalities and the timing of post-treatment assessments may also have contributed to the variability in CRP response. The differing treatment-related changes observed in IG# and CRP may therefore reflect differences in their biological determinants and response patterns, rather than greater sensitivity or superiority of IG# over CRP. Nevertheless, the persistent elevation of IG# after clinical improvement suggests that it may provide complementary information on residual systemic inflammatory burden in patients with psoriasis.

Furthermore, we assessed the potential impact of concomitant PsA on inflammatory parameters. In our cohort, no significant differences were observed between patients with and without PsA in terms of most inflammatory markers, both before and after treatment, except for higher ESR levels in the PsA group after treatment. Although PsA is generally considered to be associated with an increased systemic inflammatory burden, previous studies have reported inconsistent findings regarding its impact on hematological inflammatory markers [32,33,34]. Our results suggest that, apart from ESR, the presence of PsA may not substantially influence these parameters, and that psoriasis itself may represent a significant source of systemic inflammation regardless of joint involvement.

In the present study, IG# exhibited a weak but statistically significant correlation with PASI scores, whereas no significant correlation was identified between IG% and PASI. Previous studies in the literature have reported conflicting results regarding the relationship between inflammatory markers and psoriasis severity. While some studies have demonstrated significant positive correlations between hematological parameters such as WBC, neutrophils, NLR, and CRP and PASI scores, others have failed to show any meaningful association between indices such as NLR and platelet-to-lymphocyte and disease severity [35,36]. Similarly, in chronic inflammatory dermatologic conditions such as pemphigus, only moderate correlations have been observed between IG parameters (IG# and IG%) and disease activity, while regression analyses have suggested that IG#, rather than IG%, may serve as a more independent predictor of disease activity [25]. These discrepancies suggest that although inflammatory markers may reflect the presence of systemic inflammation in psoriasis, their role in assessing disease severity remains inconsistent. In line with this, our findings indicate that IG parameters, despite reflecting systemic inflammation, may have limited utility in accurately representing disease severity.

From a clinical standpoint, IG parameters present several advantages. They are readily accessible as part of routine complete blood count analysis, do not require additional cost or specialized assays, and can be easily integrated into daily clinical practice. In the current study, IG parameters were correlated with baseline inflammatory status, exhibited responsiveness to treatment, and remained elevated even after clinical improvement, indicating the presence of persistent systemic inflammation. These findings suggest that IG#, in particular, may be more sensitive than some conventional inflammatory markers in reflecting both treatment response and residual inflammatory burden. Consequently, IG parameters may serve as a practical and accessible biomarker for assessing systemic inflammation in patients with psoriasis; however, their clinical utility should be interpreted with caution and in conjunction with other clinical and laboratory findings.

### Limitations

The present study has several limitations. First, PASI scores were calculated by different clinicians, which may have introduced inter-observer variability in disease severity assessment. Second, the retrospective and single-center design limits the generalizability of the findings and may have introduced selection bias. Moreover, because post-treatment assessments were restricted to time points at which PASI 75 had been achieved, the observed treatment-related changes may not be generalizable to patients with an inadequate clinical response. Additionally, although patients with active infection and known chronic systemic diseases were excluded, unrecognized subclinical inflammatory conditions may have influenced hematological parameters. Furthermore, body mass index and smoking status, which may influence systemic inflammatory markers, were not systematically available and could not be evaluated. Disease duration was also not included in the analysis, which may have influenced the relationship between inflammatory markers and disease severity. Finally, the lack of subgroup analysis according to different systemic treatment modalities may represent another limitation, as various therapies may have differential effects on inflammatory parameters.

## 5. Conclusions

The present study demonstrated that both IG# and IG% were significantly higher in patients with psoriasis prior to treatment compared to healthy controls, indicating that these parameters may reflect the systemic inflammatory burden associated with psoriasis. The significant reduction in IG# following systemic treatment suggests that IG levels may be responsive to therapeutic intervention and could potentially serve as a marker for monitoring inflammatory activity and treatment response. Furthermore, the persistence of significantly elevated IG# levels in the post-treatment group compared to healthy controls may suggest the presence of ongoing subclinical systemic inflammation in psoriasis patients, even after clinical improvement. Given that IG parameters are readily obtainable from routine complete blood count analysis and represent a cost-effective and easily accessible laboratory marker, they may offer practical utility in assessing the inflammatory status in patients with psoriasis. However, these findings should be interpreted in light of the retrospective single-center design and the lack of treatment subgroup analyses. Further prospective, multicenter studies are required to validate these findings and to clarify their clinical applicability.

## Figures and Tables

**Table 1:** Comparison of laboratory parameters between pre-treatment psoriasis patients and healthy controls.

Parameter	Pre-Treatment Psoriasis Median (IQR)	Healthy Controls Median (IQR)	*p*-Value
IG# (cells/µL)	30 (20–40)	20 (10–20)	**<0.001**
IG%	0.3 (0.2–0.5)	0.3 (0.2–0.3)	**<0.001**
CRP (mg/L)	3.25 (1.48–6.33)	1.6 (0.9–2.8)	**<0.001**
WBC (cells/µL)	8180 (7110–9220)	6730 (5815–7675)	**<0.001**
Neutrophils (cells/µL)	5060 (4140–6040)	3710 (2945–4445)	**<0.001**
Lymphocytes (cells/µL)	2300 (1975–2520)	2170 (1855–2645)	0.360
Platelets (×10^3^/µL)	245 (210–304)	250 (216–293)	0.840
ESR (mm/h)	8 (6–13)	6 (5–9)	0.080
NLR	2.36 (1.89–2.70)	1.67 (1.37–2.22)	**<0.001**
SII	591 (443–728)	443 (324–559)	**<0.001**

Data are presented as median (interquartile range, IQR). Comparisons between groups were performed using the Mann–Whitney U test. Statistically significant *p* values (<0.05) are shown in bold. Abbreviations: CRP, C-reactive protein; ESR, erythrocyte sedimentation rate; IG#, immature granulocyte count; IG%, immature granulocyte percentage; NLR, neutrophil-to-lymphocyte ratio; SII, systemic immune-inflammation index; WBC, white blood cell count.

**Table 2:** Discriminatory performance of pre-treatment immature granulocyte parameters for differentiating patients with psoriasis from healthy controls.

Parameter	AUC (%95 CI)	*p* Value	Cut-Off Value	Sensitivity (%95 CI)	Specificity (%95 CI)
IG# (cells/µL)	0.697 (0.622–0.765)	**<0.001**	20	55.3 (44.1–66.1)	82.4 (72.6–89.8)
IG%	0.658 (0.581–0.73)	**<0.001**	0.3	44.4 (33.4–55.9)	80 (69.9–87.9)

Optimal cut-off values were determined using the Youden index. Statistically significant *p* values (<0.05) are shown in bold. Abbreviations: AUC, area under the receiver operating characteristic curve; CI, confidence interval; IG#, immature granulocyte count; IG%, immature granulocyte percentage.

**Table 3:** Comparison of clinical and laboratory parameters between the pre-treatment and post-treatment assessments in patients with psoriasis.

Parameter	Pre-Treatment Median (IQR)	Post-Treatment Median (IQR)	*p*-Value
PASI	12 (10.4–15.9)	0 (0–1)	**<0.001**
IG# (cells/µL)	30 (20–40)	20 (10–30)	**0.004**
IG%	0.3 (0.2–0.5)	0.3 (0.2–0.4)	0.072
CRP (mg/L)	3.25 (1.48–6.33)	2.8 (1.4–5.3)	0.160
WBC (cells/µL)	8180 (7110–9220)	7350 (6250–8890)	**<0.001**
Neutrophils (cells/µL)	5060 (4140–6040)	4050 (3240–5555)	**<0.001**
Lymphocytes (cells/µL)	2300 (1975–2520)	2350 (1965–2785)	**0.006**
Platelets (×10^3^/µL)	245 (210–304)	251 (212–302)	0.500
ESR (mm/h)	8 (6–13)	6 (5–15)	0.280
NLR	2.36 (1.89–2.70)	1.82 (1.38–2.27)	**<0.001**
SII	591 (443–728)	484 (321–626)	**<0.001**

Data are presented as median (interquartile range, IQR). Comparisons between paired measurements were performed using the Wilcoxon signed-rank test. Statistically significant *p* values (<0.05) are shown in bold. Abbreviations: CRP, C-reactive protein; ESR, erythrocyte sedimentation rate; IG#, immature granulocyte count; IG%, immature granulocyte percentage; NLR, neutrophil-to-lymphocyte ratio; PASI, Psoriasis Area and Severity Index; SII, systemic immune-inflammation index; WBC, white blood cell count.

**Table 4:** Comparison of laboratory parameters between post-treatment psoriasis patients and healthy controls.

Parameter	Post-Treatment Psoriasis Median (IQR)	Healthy Controls Median (IQR)	*p*-Value
IG# (cells/µL)	20 (10–30)	20 (10–20)	**0.005**
IG%	0.3 (0.2–0.4)	0.3 (0.2–0.3)	**0.018**
CRP (mg/L)	2.8 (1.4–5.3)	1.6 (0.9–2.8)	**0.007**
WBC (cells/µL)	7350 (6250–8890)	6730 (5815–7675)	**0.006**
Neutrophils (cells/µL)	4050 (3240–5555)	3710 (2945–4445)	0.051
Lymphocytes (cells/µL)	2350 (1965–2785)	2170 (1855–2645)	**0.034**
Platelets (×10^3^/µL)	251 (212–302)	250 (216–293)	0.870
ESR (mm/h)	6 (5–15)	6 (5–9)	0.540
NLR	1.82 (1.38–2.27)	1.67 (1.37–2.22)	0.440
SII	484 (321–626)	443 (324–559)	0.520

Data are presented as median (interquartile range, IQR). Comparisons between groups were performed using the Mann–Whitney U test. Statistically significant *p* values (<0.05) are shown in bold. Abbreviations: CRP, C-reactive protein; ESR, erythrocyte sedimentation rate; IG#, immature granulocyte count; IG%, immature granulocyte percentage; NLR, neutrophil-to-lymphocyte ratio; SII, systemic immune-inflammation index; WBC, white blood cell count.

**Table 5:** Comparison of PASI and immature granulocyte parameters according to sex in patients with psoriasis.

Parameter	Male (*n* = 51), Median (IQR)	Female (*n* = 34), Median (IQR)	*p*-Value
Pre-treatment PASI	12.5 (10.2–17.0)	12.0 (10.6–15.3)	0.490
Pre-treatment IG# (cells/µL)	20 (12.5–30)	20 (10–30)	0.410
Pre-treatment IG%	0.3 (0.2–0.4)	0.3 (0.2–0.3)	0.180
Post-treatment PASI	0 (0–0.5)	0 (0–1)	0.800
Post-treatment IG# (cells/µL)	20 (10–30)	20 (10–30)	0.980
Post-treatment IG%	0.3 (0.2–0.4)	0.3 (0.2–0.325)	0.340

Data are presented as median (interquartile range, IQR). Comparisons between male and female patients were performed using the Mann–Whitney U test. Abbreviations: PASI, Psoriasis Area and Severity Index; IG#, immature granulocyte count; IG%, immature granulocyte percentage.

## Data Availability

The data presented in this study are available on request from the corresponding author. The data are not publicly available due to privacy and ethical restrictions related to patient data.

## Supplementary Materials

The following supporting information can be downloaded at: https://www.mdpi.com/article/10.3390/jcm15166133/s1, Figure S1: Correlations between age and PASI scores and immature granulocyte parameters before and after systemic treatment.

## References

[1] Pathirana D., Ormerod A., Saiag P., Smith C., Spuls P., Nast A., Barker J., Bos J., Burmester G., Chimenti S. (2009). European S3-Guidelines on the systemic treatment of psoriasis vulgaris. J. Eur. Acad. Dermatol. Venereol..

[2] Rosenø N.A.L., Lørup E.H., Richardson C., Alarcon I., Egeberg A. (2023). Exploring disease comorbidities and temporal disease progression of psoriasis: An observational, retrospective, multi-database, cohort study. Br. J. Dermatol..

[3] Karampinis E., Papadopoulou M.M., Chaidaki K., Georgopoulou K.-E., Magaliou S., Schulze A.V.R., Bogdanos D.P., Zafiriou E. (2024). Plaque Psoriasis Exacerbation and COVID-19 Vaccination: Assessing the Characteristics of the Flare and the Exposome Parameters. Vaccines.

[4] Langley R.G., Ellis C.N. (2004). Evaluating psoriasis with Psoriasis Area and Severity Index, Psoriasis Global Assessment, and Lattice System Physician’s Global Assessment. J. Am. Acad. Dermatol..

[5] Fernandes B., Hamaguchi Y. (2007). Automated Enumeration of Immature Granulocytes. Am. J. Clin. Pathol..

[6] Maenhout T.M., Marcelis L. (2014). Immature granulocyte count in peripheral blood by the Sysmex haematology XN series compared to microscopic differentiation. J. Clin. Pathol..

[7] Senthilnayagam B., Kumar T., Sukumaran J., Jeya M., Rao K.R. (2012). Automated Measurement of Immature Granulocytes: Performance Characteristics and Utility in Routine Clinical Practice. Pathol. Res. Int..

[8] Chiang C.C., Cheng W.J., Korinek M., Lin C.Y., Hwang T.L. (2019). Neutrophils in Psoriasis. Front. Immunol..

[9] Cetın N., Kocaturk E., Tufan A.K., Kıraz Z.K., Alatas O. (2023). Immature granulocytes as biomarkers of inflammation in children with predialysis chronic kidney disease. Pediatr. Nephrol..

[10] Gupta S., Sharma N., Gupta E., Mehta S., Bhansaly P., Mehta S. (2022). Evaluation of Immature Granulocyte Count as the Earliest Biomarker for Sepsis. Indian J. Crit. Care Med..

[11] Kozanlı F., Akkök B. (2022). Contribution of immature granulocyte level to diagnosis in pleural effusion. Turk. J. Thorac. Cardiovasc. Surg..

[12] Güngör T., Özdel S., Çakici E.K., Yazilitaş F., Bağlan E., Karakaya D., Çelikkaya E., Bülbül M. (2022). An Assessment on the Effectiveness of the Immature Granulocyte Percentage in Predicting Internal Organ Involvement Among Children with Henoch-Schönlein Purpura. J. Pediatr. Hematol. Oncol..

[13] Ünal Y., Barlas A.M. (2019). The role of increased immature granulocyte percentage in the early prediction of acute necrotizing pancreatitis. Turk. J. Trauma Emerg. Surg..

[14] Incir S., Kant Calti H., Palaoglu K.E. (2020). The Role of Immature Granulocytes and Inflammatory Hemogram Indices in the Inflammation. Int. J. Med. Biochem..

[15] Bennett L., Palucka A.K., Arce E., Cantrell V., Borvak J., Banchereau J., Pascual V. (2003). Interferon and Granulopoiesis Signatures in Systemic Lupus Erythematosus Blood. J. Exp. Med..

[16] Fan S.S., Xu X., Luo Y.B., Meng X.Y., Liu Y. (2025). GM-CSF+ Th: A central player in autoimmunity. Theranostics.

[17] Karakurt G., Guven O., Aynaci E., Kerget B., Senkardesler G., Duger M. (2024). Evaluation of Hemogram Parameters in the Diagnosis of Pulmonary Embolism: Immature Granulocytes and Other New Tips. Clin. Appl. Thromb. Hemost..

[18] Yazla M., Kadıoğlu B., Demirdelen H., Aksoy F.M., Özkan E., Katipoğlu B. (2024). Predictive efficacy of immature granulocytes in acute complicated appendicitis. Rev. Assoc. Med. Bras..

[19] Üstüner M.A., Ay O.F., Özen A.V., Aydın M.C. (2025). The Role of Immature Granulocytes in Predicting Complicated Appendicitis: A Retrospective Observational Study. Ann. Ital. Chir..

[20] Topaloğlu M.S., Arpa M., Şen B., Bilgin Topaloğlu H., Cüre O. (2025). The Role of Immature Granulocytes in Fibromyalgia: An Indicator of Subclinical Inflammation?. Biomedicines.

[21] Seringec Akkececi N., Ciftcioglu M., Okyar B., Yildirim Cetin G. (2024). Relationship of immature granulocytes with disease activity in rheumatoid arthritis. Int. J. Rheum. Dis..

[22] Özcan E., Gülten S. (2023). A New Inflammation Marker in Rheumatoid Arthritis: Immature Granulocyte. Kırıkkale Üniversitesi Tıp Fakültesi Derg..

[23] Biyik Z., Yavuz Y.C., Altintepe L., Cizmecioglu A., Yakşı E., Korez M.K., Yılmaz S. (2025). Immature Granulocyte: A Novel Inflammatory Biomarker in Familial Mediterranean Fever. Int. J. Rheum. Dis..

[24] Okyar B., Yüce S., Bilen İ.H., Torun B., Öztürk İ., Çetin G.Y. (2024). Changes in immature granulocyte levels and their association with disease activation following biologic therapy in patients with ankylosing spondylitis. Reum. Clin..

[25] Kuş M.M., Yaray O.C., Öztürk P., Mülayim M.K. (2025). Can the count and percentage of immature granulocytes be used to detect disease activity in patients with pemphigus?: A preliminary study. Medicine.

[26] Xiong H., Yu Z. (2025). Association between systemic inflammation indicators and psoriasis: A cross-sectional study from NHANES. Front. Immunol..

[27] Liu Y.C., Chuang S.H., Chen Y.P., Shih Y.H. (2024). Associations of novel complete blood count-derived inflammatory markers with psoriasis: A systematic review and meta-analysis. Arch. Dermatol. Res..

[28] Demirel Öğüt N., Ayanoğlu M.A., Koç Yıldırım S., Erbağcı E., Ünal S., Gökyayla E. (2025). Are IL-17 inhibitors superior to IL-23 inhibitors in reducing systemic inflammation in moderate-to-severe plaque psoriasis? A retrospective cohort study. Arch. Dermatol. Res..

[29] Kimak-Pielas A., Robak E., Zajdel R., Żebrowska A. (2025). The Relationship Between Neutrophil-to-Lymphocyte Ratio, Platelet-to-Lymphocyte Ratio, and Systemic Immune-Inflammation Index Markers and Response to Biological Therapy in Patients with Psoriasis. Int. J. Mol. Sci..

[30] Asahina A., Umezawa Y., Yanaba K., Nakagawa H. (2016). Serum C-reactive protein levels in Japanese patients with psoriasis and psoriatic arthritis: Long-term differential effects of biologics. J. Dermatol..

[31] Paschoal R.S., Silva D.A., Cardili R.N., Souza C.d.S. (2018). Metabolic syndrome, C-reactive protein and cardiovascular risk in psoriasis patients: A cross-sectional study. Bras. Dermatol..

[32] Su Y.J. (2020). Early diagnosis of psoriatic arthritis among psoriasis patients: Clinical experience sharing. Clin. Rheumatol..

[33] Kim D.S., Shin D., Lee M.S., Kim H.J., Kim D.Y., Kim S.M., Lee M. (2016). Assessments of neutrophil to lymphocyte ratio and platelet to lymphocyte ratio in Korean patients with psoriasis vulgaris and psoriatic arthritis. J. Dermatol..

[34] Asahina A., Kubo N., Umezawa Y., Honda H., Yanaba K., Nakagawa H. (2017). Neutrophil–lymphocyte ratio, platelet–lymphocyte ratio and mean platelet volume in Japanese patients with psoriasis and psoriatic arthritis: Response to therapy with biologics. J. Dermatol..

[35] Ye J.H., Zhang Y., Naidoo K., Ye S. (2024). Neutrophil-to-lymphocyte ratio and platelet-to-lymphocyte ratio in psoriasis: A systematic review and meta-analysis. Arch. Dermatol. Res..

[36] Şener G., İnan Yuksel E., Gökdeniz O., Karaman K., Canat H.D. (2023). The Relationship of Hematological Parameters and C-reactive Protein (CRP) with Disease Presence, Severity, and Response to Systemic Therapy in Patients with Psoriasis. Cureus.

