# Supplementary material for: The Role of Immature Granulocyte Count and Percentage as Novel Indicators of Systemic Inflammation in Plaque Psoriasis
Source: J Clin Med. 2026 Aug 7;15(16):6133. doi: 10.3390/jcm15166133 (PMC13513338; doi:10.3390/jcm15166133)
Supplement: Supplementary file 1 [file jcm-15-06133-s001.zip › jcm-4432747-supplementary.pdf]

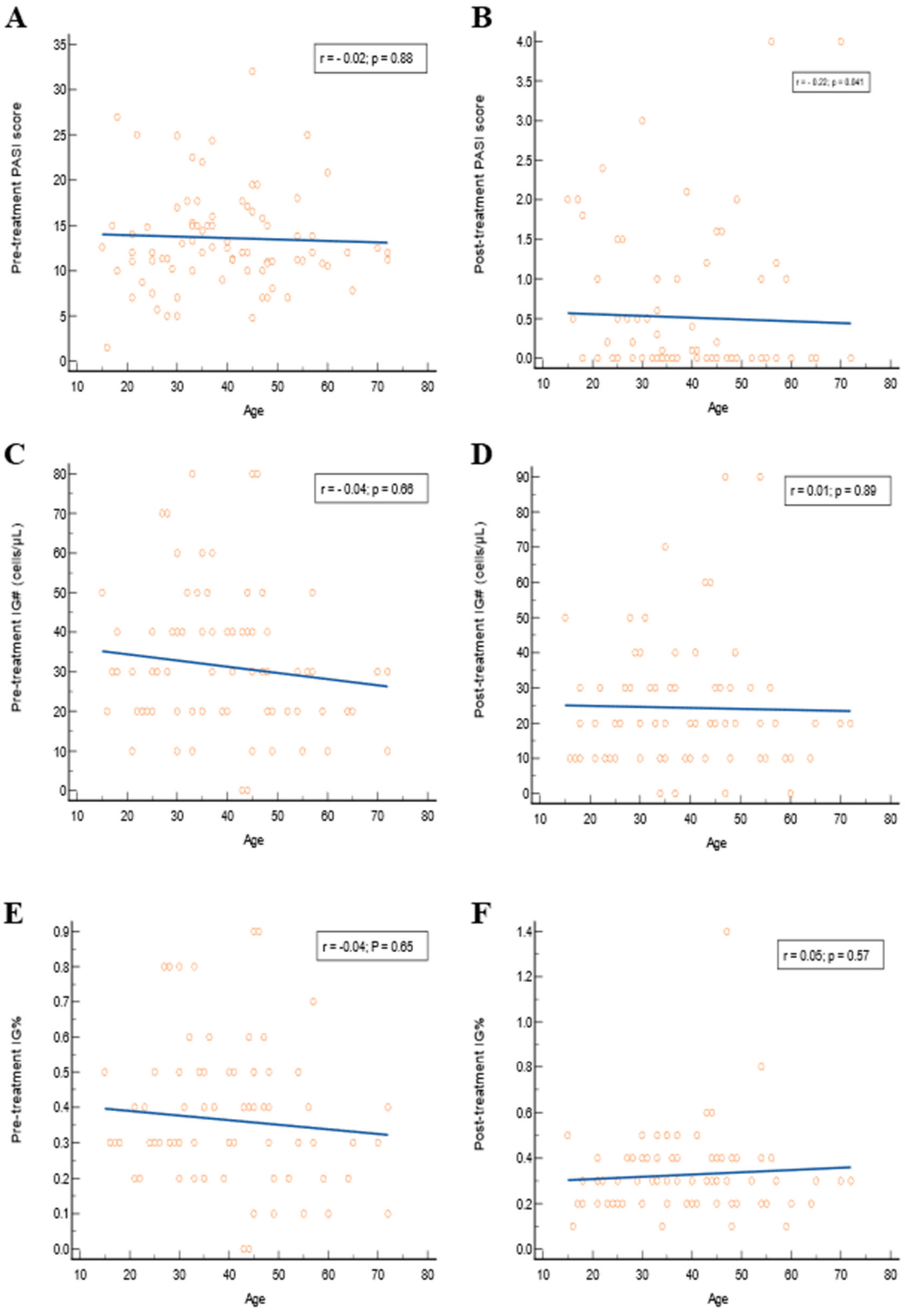

Figure S1. Correlations between age and PASI scores and immature granulocyte parameters before and after systemic treatment. (A) Age and pre-treatment PASI score; (B) age and post-treatment PASI score; (C) age and pre-treatment IG#; (D) age and post-treatment IG#; (E) age and pre-treatment IG%; and (F) age and post-treatment IG%. Correlations were assessed using Spearman's rank correlation

analysis. The circles represent individual observations. The solid lines represent the fitted linear trend lines. PASI, Psoriasis Area and Severity Index; IG#, immature granulocyte count; IG%, immature granulocyte percentage.
